# In Memoriam—Dr. Giles M. Pease

**Published:** 1892-06

**Authors:** J. M. Selfridge

**Affiliations:** Oakland, California


### IN MEMORIAM—DR. GILES M. PEASE.

Dr. Giles M. Pease was born in Boston, Mass., May 3d, 1839. His early education, like that of most American boys, was in the public schools of his native town, but he finished his literary studies in one of the academies in Boston. Just when he decided to study medicine I am not informed, but for the purpose of defraying his expenses while attending the medical college, he went upon the boards of the theatre, and at one time was Booth's best man. When sufficient means had been procured, he left the drama and commenced the study of medicine at Harvard. Before he completed his studies the war of the Rebellion broke out, and after passing a rigid examination by the United States Navy Board, standing second in his class, he enlisted in the early part of 1861 in the United States Navy as Assistant Surgeon. While packing his trunk before leaving for the seat of war, his father, Giles Pease, M. D., one of the first practitioners of Homœopathy in New England, placed a small book, entitled *Sixteen Principal Homœopathic Remedies*, and a case containing the medicines in his trunk with the remark, "they may be of use to you in emergencies." But young Giles being thoroughly indoctrinated with the teachings of Harvard, paid no attention to the little volume until his ship struck yellow fever in Southern waters. His first, second, and third patients, treated according to allopathic methods, died. Then he remembered the parting words of his father, and eagerly sought the previously-despised little book. He studied it thoughtfully, and compared the symptoms of his next patient, sick with yellow fever, with the remedies in the little book, and finally gave Arsenicum. It proved to be the simillimum, and

*cured* his patient. After this, he lost no more cases with yellow fever.

In the winter of 1862–63 his health gave way and he resigned his position in the navy and returned home. When his health was sufficiently restored, he re-entered Harvard, from which he received the degree of M. D., July 15th, 1863.

Being fired with patriotism he now enlisted in the army as Assistant Surgeon of the Fifty-fourth Regiment Massachusetts Volunteers. He soon took the place of Surgeon, and although he rapidly rose to the position of chief Medical Officer of his regiment, he was never commissioned as *full* Surgeon, owing to the prejudice against him on the part of the Surgeon-General, because of his reported leaning toward Homœopathy. In the summer of 1864, his health again failed him, and he was compelled to leave the service.

His successful experiences with the *Sixteen Principal Homœopathic Remedies* while in the service of his country, so impressed him, that on retiring from the army in the fall of 1864 he unfurled the banner of Homœopathy and commenced private practice in the city of Boston.

His opportunities in the army, and his mechanical tastes soon led him to adopt surgery as a specialty. In this branch of the profession he excelled. His boldness, his carefulness, his originality, and his successes in abdominal surgery soon won for him a *national* reputation. He was one of the *first* surgeons in the world who had the courage to discard the clamp, tie the bleeding vessels with silkworm-gut and return the stump of an ovariotomy within the abdominal wall. This was as early as 1866.

Physically, Dr. Pease was *never* a robust man. In February, 1873, he had a severe attack of pneumonia from which he made a slow and imperfect recovery. Becoming convinced that a change of climate was absolutely necessary, he left Boston, and turning his face toward the setting sun, he reached the shores of the golden State of California in the summer of 1873. The mildness of the climate and the rapid recovery of his health induced him to remain in San Francisco.

In November, 1874, "The Pacific Homœopathic Medical Society" was organized, with Dr. Pease as one of its charter members. He served as its Secretary during its entire existence with great credit to himself and the Society.

In 1877 "The California State Homœopathic Medical Society" was organized, of which Society Dr. Pease was an active member, and in 1882 he served as its Secretary, and was elected President of the Society in 1883.

For many years Dr. Pease was a valued member of "The American Institute of Homœopathy," and, had his life been spared, he would soon have reached the degree of seniority. He was also a member of "the International Hahnemannian Association," to the proceedings of which Association he contributed several papers of great practical value.

When "The Hahnemann Medical College" of San Francisco was established, he was appointed Professor of Gynæcology and Surgical Diseases of Women, which position he filled with credit to himself and honor to the School.

Dr. Pease was also an honorary member of "The Alameda County Homœopathic Medical Society," and to the interest of its meetings he was a frequent contributor, both by his personal presence and several interesting papers.

As a surgeon, and a practitioner of Homœopathy, he had no superior on the Pacific Coast. His surgical operations, especially, were marvels of neatness and mechanical exactness. He loved and honored his profession, and was jealous of its dignity. Firm in his convictions, and honest in his purposes, he was the enemy of all medical shams, come from whomsoever or from whatsoever quarter they might. Like most physicians who have received their education in allopathic colleges, he was in his earlier years of homœopathic practice a low dilutionist; but by careful observation and the study of the philosophy of the law of similars, he soon became convinced of the superiority of potentized remedies, and although on some occasions he used material doses, yet he was known among his fellows as a high dilutionist—the 200th and upward being his favorite potencies. In the study of his cases and the selection of his remedies he was what every homœopathic physician should be, very careful and painstaking. In this respect he was a model prescriber.

Although of a mechanical turn of mind, his inventions were not numerous, yet, he gave to the profession some very useful instruments.

As a writer he was clear, concise, and impressive, but, his papers were confined, almost exclusively, to the reporting of cases, many of which appeared in the medical journals.

In 1882 he published an octavo pamphlet entitled, *Gynæcological Experiences*, in which he gave his views of the medical and surgical treatment of some of the diseases which affect the womb and its appendages.

For many years he has been collecting materials for a work on *Diseases of the Kidneys and Bladder*, but, owing to failing health, and sorrows that need not be mentioned here, it was never completed.

As I have before stated, he was never a man of robust health. During the eighteen years of our acquaintance, he had frequent attacks of asthma, which during the rainy season, frequently rendered him incapable of work or study for weeks at a time.

In his case, the asthma was incurable, and I have sometimes thought it was made doubly so by his unfortunate habit—a habit, in which *no* homœopathic physician should ever indulge —the use of tobacco. He was an inveterate smoker. These attacks, frequently repeated, gradually, but surely, reduced his power of resistance, thus rendering him more and more susceptible to diseases of a graver nature.

In the winter of 1890–91 he had an attack of pleuro-pneumonia in the right lung, which nearly ended his days. He made a fairly rapid recovery, but, during the following summer, it was easy to see that he was not so well; his strength was failing. During the following December, he contracted another *severe cold*, and was again attacked with pneumonia in the left lung. He seemed to be recovering when his old enemy, the asthma, returned, and being unable to withstand the complication, he passed away from his earthly joys and sorrows, December 14th, 1891.

By some persons Dr. Pease was not understood, but those who knew him well, knew him but to admire and love him.

Peace be to his ashes.

J. M. SELFRIDGE, M. D.

OAKLAND, CALIFORNIA.

